# Determinants of Anxiety in the General Latvian Population During the COVID-19 State of Emergency

**DOI:** 10.3389/fpubh.2022.854812

**Published:** 2022-06-13

**Authors:** Jelena Vrublevska, Viktorija Perepjolkina, Kristine Martinsone, Jelena Kolesnikova, Ilona Krone, Daria Smirnova, Konstantinos N. Fountoulakis, Elmars Rancans

**Affiliations:** ^1^Department of Psychiatry and Narcology, Riga Stradins University, Riga, Latvia; ^2^Institute of Public Health, Riga Stradins University, Riga, Latvia; ^3^Faculty of Communication, Riga Stradins University, Riga, Latvia; ^4^Department of Health Psychology and Pedagogy, Riga Stradins University, Riga, Latvia; ^5^International Centre for Education and Research in Neuropsychiatry (ICERN), Samara State Medical University, Samara, Russia; ^6^Department of Psychiatry, Narcology, Psychotherapy and Clinical Psychology, Samara State Medical University, Samara, Russia; ^7^3rd Department of Psychiatry, School of Medicine, Aristotle University of Thessaloniki, Thessaloniki, Greece

**Keywords:** anxiety, COVID-19, pandemic, general population, mental health, predictors

## Abstract

**Background:**

The COVID-19 pandemic and its restrictive public health measures have seriously affected mental health of society. Social, psychological, and health-related factors have been linked to anxiety in the general population.

**Aim:**

We investigate the association of various sociopsychological and health-related determinants of anxiety and identify the predicting factors for anxiety in the general population during the COVID-19 state of emergency from in Latvia.

**Methods:**

We conducted an online survey using a randomized stratified sample of the general adult population in July 2020 for 3 weeks. Anxiety symptoms were measured using the State-Trait Anxiety Inventory (STAI-S). Sociodemographic, health-related, sociopsychological characteristics and suicidality were identified using the structured questionnaire. The statistical analysis included Pearson's chi-square test, *post hoc* analysis, and binomial logistic regression.

**Results:**

The weighted study sample included 2,608 participants. The mean STAY-S score of the total sample was 22.88 ± 12.25. In the total sample, 15.2% (*n* = 398) of participants were classified as having anxiety. The odds ratio (OR) of having anxiety was higher in females (OR = 2.44; 95% CI 1.75–3.33) and people who had experienced mental health problems in the past (OR = 1.45; 95% CI 1.03–2.04), had suicide attempt in the past (OR = 1.68; 95% CI 1.08–2.59), were worried about their health status due to COVID-19 (OR = 1.64; 95% CI 1.36–1.16), were worried about stigmatization from others if infected with COVID-19 (OR = 1.18; 95% CI 1.03–1.35), were worried about information regarding COVID-19 from the Internet (OR = 1.24; 95% CI 1.08–1.43), persons who were lonely (OR = 1.90; 95% CI 1.54–2.34), and persons with negative problem orientation (OR = 1.26; 95% CI 1.06–1.51). Protective factors were identified as having good self-rated general health (OR = 0.68, 95 % CI 0.58–0.81), maintaining a daily routine (OR = 0.74, 95 % CI 0.61–0.90), having financial stability (OR = 0.66, 95 % CI 0.55–0.79), and having good psychological resilience (OR = 0.90, 95 % CI 0.87–0.94).

**Conclusions:**

This is the first study to report a prevalence of anxiety in the general population of Latvia. Certain factors that predict anxiety, as well as protective factors were identified.

## Introduction

Even before the COVID-19 pandemic, anxiety disorders were the leading causes of burden globally, despite the existence of intervention strategies aimed at reducing their effects (1). The COVID-19 pandemic has had a significant impact on public health, including mental and physical health (2). Moreover, anxiety has been reported as a common experience among COVID-19 patients, while the public's pandemic-related health concerns and fears of contracting COVID-19 serve as contributing factors to anxiety (2, 3).

A large-scale meta-analysis of 71 published papers revealed there was a 32.6% total prevalence of anxiety during the COVID-19 pandemic (4), while the prevalence estimates of anxiety differ remarkably across countries and populations (5). Meanwhile, people with mental health disorders may be considerably more affected by emotional reactions in the form of anxiety generated by the COVID-19 pandemic (6).

Many studies have suggested that anxiety during the COVID-19 pandemic is associated with certain sociodemographic characteristics, health-related factors (e.g., mental health problems and suicidality in the past), and sociopsychological factors (e.g., loneliness, poor relationship quality, changes in daily routine and behavior, low psychological resilience, and negative problem orientation) (7–14). Available research indicates that females and those of a younger age who lived in rural areas and had lower socioeconomic status had a higher risk of anxiety (7). Moreover, other social and economic factors, such as economical struggles, unemployment, being unmarried, having chronic diseases, sedentary behavior, and poor sleep quality, were associated with anxiety during the COVID-19 pandemic (6, 15, 16). The literature has also suggested that the COVID-19 pandemic has triggered feelings of fear as a response to the sense of extreme threat for both the community and individuals (8, 17, 18). Moreover, metacognitions, intolerance of uncertainty, and emotional dysregulation have all been linked to the fear of COVID-19 and anxiety (10).

Changes in daily life, loneliness, social isolation have had a huge impact worldwide, with serious psychological implications (18, 19). Loneliness can occur not only in the context of social isolation, but can even be felt when others are physically present, and has been linked to anxiety, implying that lonely persons are more vulnerable (11, 19). Meanwhile, the prolonged “stay-at-home” and confinement conditions have led individuals to be more engaged with technology use (20). The Internet, as a valuable source of health information, has become more widely used by the general population during the ongoing COVID-19 pandemic (12). However, repeated media exposure to pandemic-related material and extensive online searches for health-related information can intensify anxiety and develop a cycle of psychological discomfort that is hard to break (12). In addition, problem-solving is a broad coping technique that promotes and sustains general competence and adaptability. It can have positive and/or negative orientations, while the deficits of positively-orientated problem-solving show significant correlations with anxiety (21, 22). Finally, resilience is a dynamic process that involves adaptation in the face of adversity and refers to the tendency to retain stable, healthy functioning following a potentially stressful life experience (23). Recent data suggest that during the COVID-19 pandemic, highly resilient, risk-tolerant individuals reported having lower anxiety (13).

There are concerns that COVID-19 pandemic could lead to increased suicide rates. However, the data concerning suicidality during the COVID-19 pandemic are not conclusive. The risk of suicide may have increased due to the stigmatization of COVID-19-infected patients and their families. Moreover, people with psychiatric illnesses may experience worsening symptoms or develop altered mental states (e.g., anxiety), which is related to increased suicide risk. High levels of suicidality have been reported previously (24), while the data on suicides from 21 countries have shown no evidence of a significant increase in suicide risk since the pandemic began (25). Conversely, other studies (14) have suggested that that the COVID-19 pandemic may trigger suicidality and behavior. For example, Fountoulakis et al. (17) assume that stress and anxiety develop first, followed by depression and suicidality.

Following the World Health Organization's (WHO) declaration of a global pandemic, the Latvian authorities declared the first state of emergency in March 2020 with a number of epidemiological security measures and restrictions, primarily the restriction of meetings, travel, most public places and educational institutions, which lasted until June 2020. Noteworthy, at that time restrictions due to the pandemic in Latvia were much milder than in other Baltic and European countries. According to the Latvian National Health Service data, as of 1 July 2020, there were 1118 confirmed COVID-19 cases in Latvia with 32 deaths and 198,508 tests having been performed. A strict lockdown due to large increase in COVID-19 cases was first introduced in October 2021 (26, 27).

Given that the COVID-19 pandemic is a global problem that has affected countries to varying degrees, there is a need for a transnational understanding of the potential sociodemographic and sociopsychological predictors of anxiety. This need is reinforced by the fact that Latvia before the pandemic had one of the highest suicide rates in Europe (28). Moreover, anxiety in the general Latvian population has not yet been estimated. In addition to determine the anxiety status of the general population during the COVID-19 pandemic, key risk and protective factors need to be identified to determine an at-risk group and measures that can be taken to protect those who are at risk from anxiety symptoms and improve their mental health.

This study aims to investigate the association between sociodemographic, health-related, and sociopsychological determinants and anxiety and identify the predicting factors for anxiety in the general population of Latvia during the state of emergency from March to June 2020.

## Methods

### The Survey

We conducted a quantitative cross-sectional online survey that included a randomized stratified sample of the Latvian general population aged 18–74 years. The survey was within the framework of the National Research Program, and a sample of the Latvian general population was a part of the COVID-19 Mental Health International for the General Population project (COMET-G) (17). COMET-G is large international study with sample of 55,589 participants from 40 countries who filled the structured questionnaire (17). The survey was translated from English into Latvian and Russian. Both translations were then studied by a Latvian- and Russian-speaking focus group for verification. The COMET-G study protocol was supplemented with sections of the questions on the socio-psychological impact of the COVID-19 pandemic and the attitude on the measures implemented by the government. The full survey consisted of 27 thematic sections, including questions on sociodemographic information, overall mental functioning, general health status, fear of COVID-19, thoughts on the preventative measures taken against COVID-19, family relationships, lifestyle changes, spiritual inquiries, Internet use, psychological resilience, emotion regulation, positive and negative orientation toward social problems, and loneliness. The detailed protocol of the COMET-G (which included questions on general data, family relationships, health status, thoughts on COVID-19 and its preventative measures, anxiety, suicidality, and lifestyle changes) is available in the web appendix at Fountoulakis et al. (17).

### Data Collection

The data collection was conducted from July 6 to 27, 2020 (29, 30). The fieldwork team that was provided by the research company KANTAR followed the ESOMAR International Code on Market and Social Research (31). The data collection was stratified by gender, age, region, urbanization, and nationality, and was based on statistics published by the Office of Citizenship and Migration Affairs of Latvia (32). A precisely selected and segmented database was used to correspond to the general population of Latvia thus ensuring the representativeness of the sample of respondents (33). An SSL (Secure Sockets Layer) data transmission protocol was used to ensure the security of the online data transmission (34). Respondents received individual invitations by e-mail, with a password and a link to an online questionnaire, which could be completed by respondents at their preferred time until the specified survey closing time July 27. A reminder about completing the questionnaire was sent to participants by email. During the fieldwork, the database was regularly cleaned. Inactive participants were deleted, and the database was continuously updated with new participants. When the respondent filled out the questionnaire, it was saved on KANTAR's server and was not available for later editing.

Each survey item was assigned an ID code, and the data were collected anonymously online. The study was approved by the Ethics Committee of Riga Stradins University, Riga, Latvia. The first page of the online questionnaire included the declaration of voluntarily consent for participation.

### Measures

#### Anxiety

Anxiety symptoms were measured using the State-Trait Anxiety Inventory (STAI-S) (35), which was part of the online questionnaire. The internal consistency of the STAI in our study was good (Cronbach's α = 0.94). The cut-off point for the STAI-S scores used in our study was based on the normative data information (mean and standard deviation scores of the non-clinical and clinical groups) (36). The cut-off score was computed as follows:


(1) $$\begin{array}{r} c=\frac{s_{0}M_{1}+s_{1}M_{0}}{s_{0}+s_{1}}, \end{array}$$

where M1 = mean of the clinical group, S1 = standard deviation of the clinical group, M0 = mean of the non-clinical group, and S0 = standard deviation of the non-clinical group (37). According to the equation, a cut-off score of 36 was determined.

The participants' changes in anxiety were assessed using self-rated responses to the question: “How much has your emotional state changed in relation to the appearance of anxiety and insecurity compared to before the COVID-19 pandemic?” The responses were scored on a five-point scale.

#### Sociodemographic Determinants

To verify the association between anxiety and the sociodemographic characteristics, the participants' gender, age, ethnicity, urbanization, family status, education, and employment were recorded. Being a close relative or caretaker of a person who is at high risk of becoming infected with COVID-19 was assessed by “yes” or “no” responses.

#### Health-Related Determinants

The participants' general health was assessed by the question: “In general, how do you rate your health over the last month?” The responses were answered on a five-point scale. There was also an additional question: “Do you suffer from any chronic medical somatic conditions (e.g., diabetes, mellitus, hypertension, asthma, etc.)?” Self-reported mental disorders in the past were acquired by the question: “In the past, have you had any mental health problem that were serious enough to make you seek professional help, psychotherapy, or medication treatment?” The responses were in the form of “yes” or “no”.

#### Suicidality and Behaviors

We used the Risk Assessment of Suicidality Scale (RASS) to assess participants' suicidality and behaviors. The RASS was previously validated in a study using a general Greek population sample and was found to be a reliable tool (38). The internal consistency of the RASS in our general Latvian population sample was found to be good (Cronbach's α = 0.93) (29).

#### Sociopsychological Determinants

We assessed fear of COVID-19, relationship quality, religious/spiritual inquiries, Internet use, and daily routine using the questions that are available on the COMET-G's web appendix (17).

#### Psychological Determinants

We evaluated loneliness using the statement: “I felt lonely more often during the state of emergency situation than in the situation before.” The responses were scored on a four-point scale. We used the Emotion Regulation Skills Questionnaire (ERSQ-27), which was previously adapted for use in Latvia (39–41), to evaluate participants' emotional regulation ability. The ERSQ consists of 27 statements divided into 9 scales, with responses scored on a five-point scale. However, this study only used the total score (Cronbach's α = 0.96). We used the Psychological Resilience Scale, which is a seven-item measure (Cronbach's α = 0.87), to assess participants' psychological resilience. The responses were scored on a five-point scale (42).

Finally, we used the Social Problem-Solving Inventory-Revised Version (SPSI-R) (43), which was previously adapted for use in Latvia (44, 45), and is a multidimensional measure containing 52 statements. This study used two short-form scales: the Negative Problem Orientation (NPO) (Cronbach's α = 0.87) and the Positive Problem Orientation (PPO) (Cronbach's α = 0.85). The responses were scored on a five-point scale.

### Statistical Analysis

Descriptive statistics were computed for all variables used in the analyses. A cut-off point of the STAI-S score (≥ 36) was used to determine anxiety. We conducted between-group comparisons of frequencies using Pearson's chi-square test for categorical variables, and the *post hoc* analysis involved pairwise comparisons using the multiple z-test of two proportions with a Bonferroni correction. An independent samples *t*-test was used to analyze the mean differences for the continuous variables between anxiety and non-anxiety group. Variables that achieved a screening level of significance (*p* < 0.05) were simultaneously entered into a binomial logistic regression. Data were analyzed with SPSS version 27.0.

## Results

### Sociodemographic Determinants and Their Association With Anxiety

Of the 3,110 questionnaires received, after data cleaning and weighing 2,608 questionnaires were obtained. The mean STAI-S score of the total sample is 22.88 ± 12.25. In the total sample, 15.2% (*n* = 398) are classified as having anxiety. Table 1 presents the sample's sociodemographic characteristics and a chi-square test results. All expected cell frequencies were greater than five. The prevalence of anxiety among females is much higher than among males (77.1 vs. 22.9%, respectively). The comparison by age group reveals that the proportion of 18–29-year-olds is significantly higher in the anxiety group compared to the non-anxiety group (21.9 vs. 12.6%, respectively) and lower in the age group containing 40–49-year-olds (17.8 vs. 22.6%, respectively). The anxiety group has a difference in the proportion of Latvians and Russians (60.8 vs. 32.2%, respectively) when compared to the non-anxiety group (67.1 vs. 26.7%, respectively). The proportion of people living in the rural area is lower in the anxiety group compared to the non-anxiety group (22.1 vs. 27.6%, respectively). Meanwhile, those who are caretakers or close relatives of a person in a vulnerable group are more likely to meet the criteria of having anxiety compared to participants who are not (46.7 vs. 34.4%, respectively). The results are statistically significant (*p*-values are displayed in Table 1). There was not a statistically significant association between anxiety and such sociodemographic variables as family status [χ^2^(3) = 2.84, *p* = 0.416], education [χ^2^(2) = 2.89, *p* = 0.235], and employment [χ^2^(3) = 3.68, *p* = 0.298].

**Table 1:** Sociodemographic determinants and their association with anxiety (*n* = 2,608).

**Characteristics**	**All respondents (*N* = 2,608)**	**Anxiety below cut-off point (*n* = 2,210)**	**Anxiety above cut-off point (*n* = 398)**
Gender		χ^2^ = 55.95, *p* < 0.0001	
Male	1,036 (39.7%)	945 (42.8%)_a_	91 (22.9%)_b_
Female	1,570 (60.2%)	1,263 (57.2%)_a_	307 (77.1%)_b_
Other/ did not want to define	2 (0.1%)	–	–
Age		χ^2^ = 31.75, *p* < 0.0001	
18–29	365 (14.0%)	278 (12.6%)_a_	87 (21.9%)_b_
30–39	538 (20.6%)	446 (20.2%)_a_	92 (23.1%)_a_
40–49	570 (21.9%)	499 (22.6%)_a_	71 (17.8%)_b_
50–59	598 (22.9%)	513 (23,2%)_a_	85 (21.4%)_a_
60–69	433 (16.6%)	380 (17.2%)_a_	53 (13.3%)_a_
70 and older	104 (4.0%)	94 (4.3%)_a_	10 (2.5%)_a_
Ethnicity		χ^2^ = 5.96, *p* = 0.051	
Latvian	1,724 (66.1%)	1,482 (67.1%)_a_	242 (60.8%)_b_
Russian	719 (27.6 %)	591 (26.7%)_a_	128 (32.2%)_b_
Other	165 (6.3%)	137 (6.2%)_a_	28 (7.0%)_a_
Urbanization		χ^2^ = 5.53, *p* = 0.063	
Capital city	1,013 (38.8%)	844 (38.2%)_a_	169 (42.5%)_a_
Other city or town	897 (34.4%)	756 (34.2%)_a_	141 (35.4%)_a_
Rural area	698 (26.8%)	610 (27.6%)_a_	88 (22.1%)_b_
Family status		χ^2^ = 2.84, *p* = 0.416	
Single	469 (18.0%)	386 (17.5%)_a_	83 (20.9%)_a_
Married or in relationship	1,733 (66.4%)	1,480 (67.0%)_a_	253 (63.6%)_a_
Divorced/widowed	371 (14.2%)	315 (14.3%)_a_	56 (14.1%)_a_
Other	35 (1.3%)	29 (1.3%)_a_	6 (1.5%)_a_
Education		χ^2^ = 2.89, *p* = 0.235	
Less than high school degree	62 (2.4%)	57 (2.6%)_a_	5 (1.3%)_a_
High school degree	964 (37.0%)	810 (36.7%)_a_	154 (38.7%)_a_
More than high school degree	1,544 (59.2%)	1,311 (59.4%)_a_	233 (58.5%)_a_
Missing data/ unknown	38 (1.5%)	32 (1.4%)_a_	6 (1.5%)_a_
Employment		χ^2^ = 3.68, *p* = 0.298	
Employed	1,873 (71.8%)	1,598 (72.3%)_a_	275 (69.1%)_a_
Unemployed	197 (7.6%)	158 (7.2%)_a_	39 (9.8%)_a_
Economically inactive (retired, student, housewife, etc.)	498 (19.1%)	420 (19.0%)_a_	78 (19.6%)_a_
Other	40 (1.5%)	34 (1.5%)_a_	6 (1.5%)_a_
B4 Caretaker or close relative of a person that belongs to a vulnerable group		χ^2^ = 22.06, *p* < 0.0001	
Yes	947 (36.3%)	761 (34.4%)_a_	186 (46.7%)_b_
No	1,661 (63.7%)	1,449 (65.6%)_a_	212 (53.3%)_b_

*Each subscript letter denotes a subset of Anxiety score (below/above cut-off point) categories whose column proportions do not differ significantly from each other at the 0.05 level*.

### Health-Related Determinants of Anxiety

All health-related variables analyzed in this study were statistically significantly associated with anxiety (Table 2). Results of chi-square test show that of those who had anxiety, 61.3% show that their emotional state has worsened a little compared to 23.0% of those without anxiety, and 17.3% show that “It got a lot worse” compared to 0.9% of the group without anxiety. A total of 13.1% of respondents with anxiety state that their anxiety is “Neither better nor worse” compared to 71.4% of participants without symptoms of anxiety. A total of 35.9% of respondents with anxiety report a moderate or bad general health status compared to 11.2% of respondents without any health conditions. A total of 34.7% of those with anxiety suffer from chronic somatic conditions compared to 27.1% of respondents without anxiety. The participants with anxiety also have had significantly more mental health disorders in the past.

**Table 2:** Health-Related determinants of anxiety (*n* = 2,608).

**Characteristics**	**All respondents (*N* = 2,608)**	**Anxiety below cut-off point (*n* = 2,210)**	**Anxiety above cut-off point (*n* = 398)**
F21. How much has your emotional state changed in relation to the appearance of anxiety and insecurity compared to before the COVID-19 epidemic? (*M* = 2.71, *SD* = 0.66)		χ^2^ = 636.42, *p* < 0.0001	
It got a lot worse	88 (3.4%)	19 (0.9%)_a_	69 (17.3%)_b_
It got a little worse	751 (28.8%)	507 (23.0%)_a_	244 (61.3%)_b_
Neither better nor worse	1,631 (62.5%)	1,577 (71.4%)_a_	52 (13.1%)_b_
It's a little improved	106 (4.1%)	79 (3.6%)_a_	27 (6.8%)_b_
It has improved a lot	32 (1.2%)	26 (1.2%)_a_	6 (1.5%)_a_
B1. In general, your health over the last month can be described as (*M* = 2.59, *SD* = 0.98)		χ^2^ = 179.28, *p* < 0.0001	
Excellent	425 (16.3%)	399 (18.1%)_a_	26 (6.5%)_b_
Very good	665 (25.5%)	596 (27.0%)_a_	69 (17.3%)_b_
Good	1,127 (43.2%)	967 (43.8%)_a_	160 (40.2%)_a_
Moderate	333 (12.8%)	214 (9.7%)_a_	119 (29.9%)_b_
Bad	58 (2.2%)	34 (1.5%)_a_	24 (6.0%)_b_
B2. Do you suffer from any chronic medical somatic condition (for example: diabetes, mellitus, hypertension, asthma, etc.)?		χ^2^ = 9.65, *p* < 0.002	
Yes	736 (28.2%)	598 (27.1%)_a_	138 (34.7%)_b_
No	1,872 (71.8%)	1,612 (72.9%)_a_	260 (65.3%)_b_
B5. In the past, have you had any mental health problem serious enough to make you seek professional health, psychotherapy or medication treatment?		χ^2^ = 123.98, *p* < 0.0001	
Yes	410 (15.7%)	273 (12.4%)_a_	137 (34.4%)_b_
No	2,198 (84.3%)	1,935 (87.6%)_a_	261 (65.6%)_b_
Anxiety in the past		χ^2^ = 108.71, *p* < 0.0001	
Yes	217 (8.3%)	131 (5.9%)_a_	86 (21.6%)_b_
No	2,391 (91.7%)	2,077 (94.1%)_a_	312 (78.4%)_b_
Depression in the past		χ^2^ = 82.74, *p* < 0.0001	
Yes	220 (8.4%)	140 (6.3%)_a_	80 (20.1%)_b_
No	2,388 (91.6%)	2,068 (93.7%)_a_	318 (79.9%)_b_
Psychosis in the past		χ^2^ = 13.79, *p* < 0.0001	
Yes	27 (1.0%)	16 (0.7%)_a_	11 (2.8%)_b_
No	2,581 (99.0%)	2,192 (99.3%)_a_	387 (97.2%)_b_
Bipolar Disorder		χ^2^ = 11.25, *p* < 0.001	
Yes	12 (0.5%)	6 (0.3%)_a_	6 (1.5%)_b_
No	1,596 (99.5%)	2,204 (99.7%)_a_	392 (98.5%)_b_
Other		χ^2^ = 13.84, *p* < 0.0001	
Yes	50 (1.9%)	33 (1.5%)_a_	17 (4.3%)_b_
No	2,558 (98.1%)	2,177 (98.7%)_a_	381 (95.7%)_b_

*Each subscript letter denotes a subset of Anxiety score (below/above cut-off point) categories whose column proportions do not differ significantly from each other at the 0.05 level*.

Table 3 shows that 20.9% of the participants who have anxiety confirm that they have a fear of dying, 2.6% have frequent thoughts of harming themselves, and 3.6% have suicide ideation. Participants with anxiety show an increased tendency to think about suicide compared to those without anxiety (15.3 vs. 4.1%, respectively). A total of 11% of participants with anxiety indicated at least one attempted suicide in the past compared to 4.8% of participants without anxiety.

**Table 3:** The association of anxiety and suicidality and self-harm history in the general population of Latvia during the COVID-19 state of emergency (*n* = 2,608).

**Characteristics**	**All respondents (*N* = 2,608)**	**Anxiety below cut-off point (*n* = 2,210)**	**Anxiety above cut-off point (*n* = 398)**
O1. Are you afraid that you are going to die? (*M* = 1.37, *SD* = 0.62)		χ^2^ = 360.70, *p* < 0.0001	
Not at all	1,804 (69.2%)	1,662 (75.2%)_a_	142 (35.7%)_b_
A little bit	666 (25.5%)	493 (22.3%)_a_	173 (43.5%)_b_
Much	106 (4.1%)	48 (2.2%)_a_	58 (14.6%)_b_
Very much	32 (1.2%)	7 (0.3%)_a_	25 (6.3%)_b_
O5. Do you think of harming yourself psychically? (*M* = 1.07, *SD* = 0.32)		χ^2^ = 45.40, *p* < 0.0001	
Not at all	2,472 (94.8%)	2,118 (95.8%)_a_	354 (88.9%)_b_
A little bit	105 (4.0%)	71 (3.2%)_a_	34 (8.5%)_b_
Much	20 (0.8%)	17 (0.8%)_a_	3 (0.8%)_a_
Very much	11 (0.4%)	4 (0.2%)_a_	7 (1.8%)_b_
O6. Do you often think of committing suicide if you have the chance? (*M* = 1.07, *SD* = 0.32)		χ^2^ = 43.17, *p* < 0.0001	
Not at all	2,479 (95.1%)	2,123 (96.1%)_a_	356 (89.4%)_b_
A little bit	96 (3.7%)	68 (3.1%)_a_	28 (7.0%)_b_
Much	23 (0.9%)	16 (0.7%)_a_	7 (1.8%)_b_
Very much	10 (0.4%)	3 (0.1%)_a_	7 (1.8%)_b_
O11. How much has your tendency to thing about death and/or suicide changed, compared to before outbreak of COVID-19? (*M* = 3.07, *SD* = 0.59)		χ^2^ = 80.51, *p* < 0.0001	
Very much increased	32 (1.2%)	20 (0.9%)_a_	12 (3.0%)_b_
Increased a bit	119 (4.6%)	70 (3.2%)_a_	49 (12.3%)_b_
Neither increased, nor decreased	2,260 (86.7%)	1,956 (88.5%)_a_	304 (76.4%)_b_
Decreased a bit	40 (1.5%)	32 (1.4%)_a_	8 (2.0%)_a_
Very much decreased	157 (6.0%)	132 (6.0%)_a_	25 (6.3%)_a_
O12. Have you ever hurt yourself in any way deliberately, during your whole life so far? (*M* = 1.14, *SD* = 0.51)		χ^2^ = 13.81, *p* < 0.003	
Never	2,375 (91.1%)	2,030 (91.9%)_a_	345 (86.7%)_b_
Once	129 (4.9%)	103 (4.7%)_a_	26 (6.5%)_a_
2–3 times	69 (2.6%)	53 (2.4%)_a_	16 (4.0%)_a_
Many times	35 (1.3%)	24 (1.1%)_a_	11 (2.8%)_b_
O13. Have you ever attempted suicide, during your whole life so far? (*M* = 1.08, *SD* = 0.33)		χ^2^ = 28.74, *p* < 0.0001	
Never	2,457 (94.2%)	2,103 (95.2%)_a_	354 (88.9%)_b_
Once	111 (4.3%)	79 (3.6%)_a_	32 (8.0%)_b_
2–3 times	33 (1.3%)	25 (1.1%)_a_	8 (2.0%)_a_
Many times	7 (0.3%)	3 (0.1%)_a_	4 (1.0%)_b_

*Each subscript letter denotes a subset of Anxiety score (below/above cut-off point) categories whose column proportions do not differ significantly from each other at the 0.05 level*.

### Sociopsychological Determinants of Anxiety

Table 4 shows that moderate and severe fears of contracting COVID-19 are statistically significantly more prevalent in participants with anxiety than those without anxiety (63.8 vs. 27.3%, respectively) as well as the fear that a family member could contract COVID-19 and die (50.6 vs. 15.4%, respectively). Meanwhile, fear of possible stigmatization (i.e., in the case of contracting COVID-19, people would distance themselves from the infected person and behave differently to them) are statistically significantly associated with those with anxiety than those without anxiety (64.3 vs. 36.6%, respectively). The belief that the COVID-19 precautions are effective is not associated with symptoms of anxiety.

**Table 4:** The association of anxiety and fears, thoughts about COVID-19, and religious/spiritual inquiries in the general population of Latvia during the COVID-19 state of emergency (*n* = 2,608).

**Characteristics**	**All respondents (*N* = 2,608)**	**Anxiety below cut-off point (*n* = 2,210)**	**Anxiety above cut-off point (*n* = 398)**
C1. Are you afraid that you will contract the coronavirus? (*M* = 2.19, *SD* = 0.96)		χ^2^ = 455.57, *p* < 0.0001	
Never	649 (24.9%)	621 (28.1%)_a_	28 (7.0%)_b_
A little	1,102 (42.3%)	986 (44.6%)_a_	116 (29.1%)_b_
Moderately	624 (23.9%)	508 (23.0%)_a_	116 (29.1%)_b_
Much	176 (6.7%)	83 (3.8%)_a_	93 (23.4%)_b_
Very much	57 (2.2%)	12 (0.5%)_a_	45 (11.3%)_b_
C2. Do you believe that the precautions work effectively or that if you are about to contract the disease, you will contract it anyway? (*M* = 1.18, *SD* = 0.39)		χ^2^ = 1.34, *p* = 0.248	
Precautions work effectively	2,131 (81.7%)	1,814 (82.1%)_a_	317 (79.6%)_a_
Precautions cannot protect you	477 (18.3%)	396 (17.9%)_a_	81 (20.4%)_a_
C3. Does the possibility that a member of your family could contract the coronavirus and die because of it makes you frightened? (*M* = 2.52, *SD* = 1.15)		χ^2^ = 312.00, *p* < 0.0001	
Never	512 (19.6%)	494 (22.4%)_a_	18 (4.5%) _b_
A little	930 (35.7%)	836 (37.8%)_a_	94 (23.6%)_b_
Moderately	626 (24.0%)	541 (24.5%)_a_	85 (21.4%)_a_
Much	371 (14.2%)	256 (11.6%)_a_	115 (28.9%)_b_
Very much	169 (6.5%)	83 (3.8%)_a_	86 (21.6%)_b_
C4. Are you afraid that in case you contract the coronavirus, some people will step away from your life and behave to you in a different way later? (*M* = 1.70, *SD* = 1.00)		χ^2^ = 224.54, *p* < 0.0001	
Never	1,544 (59.2%)	1,402 (63.4%)_a_	142 (35.7%)_b_
A little	550 (21.1%)	457 (20.7%)_a_	93 (23.4%)_a_
Moderately	333 (12.8%)	259 (11.7%)_a_	74 (18.6%)_b_
Much	127 (4.9%)	70 (3.2%)_a_	57 (14.3%)_b_
Very much	54 (2.1%)	22 (1.0%)_a_	32 (8.0%)_b_
P1. Over the last 2–3 weeks, my religious/ spiritual inquiries have been increased (*M* = 1.27, *SD* = 0.56)		χ^2^ = 44.36, *p* < 0.0001	
Not at all	2,023 (77.6%)	1,761 (79.7%)_a_	262 (65.8%)_b_
A little bit	475 (18.2%)	373 (16.9%)_a_	102 (25.6%)_b_
Much	89 (3.4%)	60 (2.7%)a	29 (7.3%)_b_
Very much	21 (0.8%)	16 (0.7%)a	5 (1.3%)_a_

*Each subscript letter denotes a subset of Anxiety score (below/above cut-off point) categories whose column proportions do not differ significantly from each other at the 0.05 level*.

Table 5 shows that moderate to severe worries about COVID-19 information on the Internet are statistically significantly more prevalent in respondents with anxiety than those without anxiety (50.0 vs. 27.4%, respectively). Participants with anxiety are more prone to using the Internet moderately to more than usual than the participants without anxiety (59.3 vs. 34.1%, respectively). Increased use of social media is also associated with the tendency to meet the criteria of anxiety (46.5 vs. 21.4%, respectively). The results show statistical significance.

**Table 5:** The association of anxiety and Internet use characteristics in the general population of Latvia during the COVID-19 state of emergency (*n* = 2,608).

**Characteristics**	**All respondents (*N* = 2,608)**	**Anxiety below cut-off point (*n* = 2,210)**	**Anxiety above cut-off point (*n* = 398)**
K1. The information and use of the internet worry me about the issue regarding the COVID-19 (*M* = 1.94, *SD* = 0.99)		χ^2^ = 130.98, *p* < 0.0001	
Not at all	1,059 (44.4%)	1,063 (48.1%)_a_	96 (24.1%)_b_
A little	640 (24.5%)	537 (24.3%)_a_	103 (25.9%)_a_
Moderately	657 (25.2%)	518 (23.4%)_a_	139 (34.9%)_b_
Much	122 (4.7%)	75 (3.4%)_a_	47 (11.8%)_b_
Very much	30 (1.2%)	17 (0.6%)_a_	13 (3.3%)_a_
K2. Generally, most of the internet sources regarding information about COVID-19 are misinforming/ misleading (*M* = 2.68, *SD* = 1.09)		χ^2^ = 5.22, *p* = 0.266	
Not at all	378 (14.5%)	320 (14.5%)_a_	58 (14.6%)_a_
A little	822 (31.5%)	703 (31.8%)_a_	119 (29.9%)_a_
Moderately	828 (31.7%)	705 (31.9%)_a_	123 (30.9%)_a_
Much	425 (16.3%)	346 (15.7%)_a_	79 (19.8%)_b_
Very much	155 (5.9%)	136 (6.2%)_a_	19 (4.8%)_a_
K3. Due to the conditions, the internet takes up more of my time than usual (*M* = 2.21, *SD* = 1.26)		χ^2^ = 142.72, *p* < 0.0001	
Not at all	1,084 (41.6%)	992 (44.9%)_a_	92 (23.1%)_b_
A little	536 (20.6%)	466 (21.1%)_a_	70 (17.6%)_a_
Moderately	468 (17.9%)	384 (17.4%)_a_	84 (21.1%)_a_
Much	391 (15.0%)	293 (13.3%)_a_	98 (24.6%)_b_
Very much	129 (4.9%)	75 (3.4%)_a_	54 (13.6%)_b_
K4. How much do you use the social media while in insolation at home? (*M* = 1.80, *SD* = 0.51)		χ^2^ = 112.83, *p* < 0.0001	
More than before	658 (25.2%)	473 (21.4%)_a_	185 (46.5%)_b_
The same as before	1,817 (69.7%)	1,621 (73.3%)_a_	196 (49.2%)_b_
Less than before	133 (5.1%)	116 (5.2%)_a_	17 (4.3%)_a_

*Each subscript letter denotes a subset of Anxiety score (below/above cut-off point) categories whose column proportions do not differ significantly from each other at the 0.05 level*.

Table 6 shows that increased conflicts with family members (17.6% of those with anxiety vs. 4.2% of those without anxiety), worsening of the overall quality of relationships with the family members (16.3% of those with anxiety vs. 3.6% of those without anxiety), difficulties in maintaining a basic daily routine (49.7% of those with anxiety vs. 20.8% of those without anxiety), financial difficulties due to the pandemic (50% of those with anxiety vs. 28.9% of those without anxiety), and feelings of loneliness (64% of those with anxiety vs. 24.8% of those without anxiety) show statistically significant association with anxiety.

**Table 6:** The association of anxiety and quality of relationships, daily routine and financial difficulties during COVID-19 epidemic (*n* = 2,608).

**Characteristics**	**All respondents (*N* = 2,608)**	**Anxiety below cut-off point (*n* = 2,210)**	**Anxiety above cut-off point (*n* = 398)**
E3. Are there any conflicts with the rest of your family members during this period? (*M* = 2.71, *SD* = 0.77)		χ^2^ = 109.36, *p* < 0.0001	
Much less	299 (11.5%)	252 (11.4%)_a_	47 (11.8%)_a_
Less	351 (13.5%)	304 (13.8%)_a_	47 (11.8%)_a_
Same	1,796 (68.9%)	1,562 (70.7%)_a_	234 (58.8%)_b_
More	143 (5.5%)	84 (3.8%)_a_	59 (14.8%)_b_
Much more	19 (0.7%)	8 (0.4%)_a_	11 (2.8%)_b_
E4. Has the overall quality of relationships with the other members of your family changed compared to the one before the quarantine, due to COVID-19? (*M* = 3.07, *SD* = 0.48)		χ^2^ = 110.63, *p* < 0.0001	
Much worse	11 (0.4%)	7 (0.3%)_a_	4 (1.0%)_a_
Worse	133 (5.1%)	72 (3.3%)_a_	61 (15.3%)_b_
It has not changed	2,165 (83.0%)	1,885 (85.3%)_a_	280 (70.4%)_b_
A little bit better	257 (9.9%)	211 (9.5%)_a_	46 (11.6%)_a_
Much better	42 (1.6%)	35 (1.6%)_a_	7 (1.8%)_a_
E5. Do you manage to maintain a basic daily routine (waking up in the morning, regular meals and sleeping hours, activities) both yourself (if you live alone) or as a family? (*M* = 2.76, *SD* = 0.72)		χ^2^ = 153.31, *p* < 0.0001	
Not at all	203 (7.8%)	136 (6.2%)_a_	67 (16.8%)_b_
Somehow, but not always	453 (17.4%)	322 (14.6%)_a_	131 (32.9%)_b_
Generally, yes	1,708 (65.5%)	1,537 (69.5%)_a_	171 (43.0%)_b_
Clearly follow (or adhere to) a routine	244 (9.4%)	215 (9.7%)_a_	29 (7.3%)_a_
E7. How are your finances as a result of the outbreak? (*M* = 2.67, *SD* = 0.76)		χ^2^ = 99.44, *p* < 0.0001	
Much more difficult than before	229 (8.8%)	149 (6.7%)_a_	80 (20.1%)_b_
Somehow more difficult	610 (23.4%)	491 (22.2%)_a_	119 (29.9%)_b_
Same as always	1,584 (60.7%)	1,404 (63.5%)_a_	180 (45.2%)_b_
Somehow easier	163 (6.3%)	147 (6.7%)_a_	16 (4.0%)_b_
Much easier than before	22 (0.8%)	19 (0.9%)_a_	3 (0.8%)_a_
Loneliness (*M* = 1.40, *SD* = 0.68)		χ^2^ = 358.18, *p* < 0.0001	
Not at all	1,804 (69.2%)	1,661 (75.2%)_a_	143 (35.9%)_b_
Somewhat	614 (23.5%)	459 (20.8%)_a_	155 (38.9%)_b_
Moderately so	139 (5.3%)	78 (3.5%)_a_	61 (15.3%)_b_
Very much so	51 (2.0%)	12 (0.5%)_a_	39 (9.8%)_b_

*Each subscript letter denotes a subset of Anxiety score (below/above cut-off point) categories whose column proportions do not differ significantly from each other at the 0.05 level*.

### Psychological Determinants of Anxiety

Table 7 shows that psychological factors such as resilience, emotional regulation skills, and social problem-solving skills (such as positive and negative problem orientation) show a weak to moderate association with the STAI-S score. Participants with anxiety show significantly lower results for psychological resilience (large effect size), emotional regulation skills, and positive problem orientation (small effect size for both variables), but higher mean scores for negative problem orientation (large effect size).

**Table 7:** Descriptive statistics of psychological characteristics and *t*-test results in the general population of Latvia during the COVID-19 state of emergency (*n* = 2,608).

**Variable**	**All respondents** **(*****N*** **=** **2,608)**		**Anxiety below cut-off point** **(*****n*** **=** **2,210)**		**Anxiety above cut-off point** **(*****n*** **=** **398)**		***t***	***Cohen's d***
	***M***	***SD***	***M***	***SD***	***M***	***SD***		
Psychological Resilience	25.56	4.91	26.18	4.55	22.11	5.40	14.19***	0.81
Successful emotion regulation	2.48	0.73	2.50	0.74	2.38	0.67	3.23**	0.17
Negative problem orientation	1.30	0.86	1.20	0.81	1.85	0.91	−13.29***	0.75
Positive problem orientation	2.19	2.18	2.21	0.86	2.07	0.81	2.93**	0.17

**p <0.01. ***p <0.001. The results of t-test (assuming unequal variances) comparing the parameter estimates between the two groups (no-clinically significant anxiety, Anxiety below cut-off point; and clinically significant anxiety, Anxiety above cut-off point).

### Factors That Predict Anxiety

A binomial logistic regression was performed to ascertain the effects of socio-demographic, health-related, life-style and psychological variables on the likelihood that participants have anxiety. Twenty-eight factors which were found to be associated with anxiety at a *p*-value of <0.05 were further analyzed using the multiple logistic regression model to determine the predictors of anxiety. Linearity of the continuous variables (gender, B1, O1, O5, O6, O11, O12, O13, C1, C3, C4, P1, E3, E4, E5, E7, K1, K3, K4, Loneliness, Emotion Regulation, Psychological Resilience, NPO and PPO) with respect to the logit of the dependent variable was assessed *via* the Box-Tidwell procedure (46). A Bonferroni correction was applied using all 50 terms in the model resulting in statistical significance being accepted when *p* < 0.001 (47). Based on this assessment, all continuous independent variables were found to be linearly related to the logit of the dependent variable. There was 66 standardized residuals with a value of 10.82 to −7.11 (M = 3.66, SD = 2.89) standard deviations, which all were kept in the analysis, because they form only 2.53% of the total sample, and 61 of them (92.42%) are participants of the anxiety group, which is of our interest and represent real cases. The logistic regression model was statistically significant [χ^2^ (28) = 882.87, *p* < 0.001], explained 50.0% (*R*^2^) of the variance in clinically-significant anxiety, and correctly classified 89.8% of cases. Of the 28 predictor variables for anxiety, the following 15 were statistically significant and are presented in Table 8: gender (female: OR = 2.44, 95 % CI 1.75–3.33, *p* < 0.001), having mental health problems in the past (OR = 1.45, 95 % CI 1.03–2.04, *p* = 0.035), fear of dying during the state of emergency (OR = 1.67, 95 % CI 1.33–2.10, *p* < 0.001), suicidality in the past (OR = 1.68, 95 % CI 1.08–2.59, *p* = 0.020), fear about one's health status due to COVID-19 (OR = 1.64, 95 % CI 1.36–1.97, *p* < 0.001), fear about a family member's health due to COVID-19 (OR = 1.36, 95 % CI 1.16–1.58, *p* < 0.001), fear of stigmatization if infected with COVID-19 (OR = 1.18, 95 % CI 1.03–1.35, *p* = 0.016), worried about information regarding COVID-19 from the Internet (OR = 1.24, 95 % CI: 1.08–1.43, *p* = 0.003), loneliness (OR = 1.90, 95 % CI: 1.54–2.34, *p* < 0.001), and negative problem orientation (OR = 1.26, 95 % CI 1.06–1.51, *p* = 0.011).

**Table 8:** Logistic regression predicting likelihood of anxiety based on sociodemographic, health-related, relationship quality, daily routine, internet use and psychological characteristics in the general population of Latvia during the COVID-19 state of emergency.

	***B***	**S.E**.	**Wald**	***df***	***p***	**Odds Ratio**	**95% CI for Odds Ratio**	
							**Lower**	**Upper**
Age	−0.01	0.01	1.75	1	0.185	0.99	0.98	1.00
Gender (female *vs*. male)	0.89	0.17	27.73	1	0.000	2.44	1.75	3.33
B1. General health status	−0.38	0.08	20.27	1	0.000	0.68	0.58	0.81
B2. Chronic medical somatic condition (yes *vs*. no)	0.01	0.17	0.001	1	0.977	1.01	0.72	1.40
B4. Caretaker of person from vulnerable group (yes *vs*. no)	0.25	0.15	2.93	1	0.087	1.29	0.96	1.72
B5. Mental health problems in the past (yes *vs*. no)	0.37	0.18	4.46	1	0.035	1.45	1.03	2.04
O1. Fear to die during the emergency state	0.51	0.12	19.74	1	0.000	1.67	1.33	2.10
O5. Thoughts of harming oneself during the emergency state	−0.55	0.28	3.98	1	0.050	0.58	0.34	0.99
O6. Thoughts about death/suicide during the emergency state	0.16	0.29	0.31	1	0.576	1.18	0.67	2.08
O11. Changes in the frequency of thoughts about death/suicide during the emergency state	0.08	0.12	0.41	1	0.524	0.93	0.74	1.17
O12. Self-harm in the past	−0.30	0.16	3.62	1	0.057	0.74	0.547	1.01
O13. Suicide attempt in the past	0.52	0.22	5.39	1	0.020	1.68	1.08	2.59
C1. Fear about one's health due to coronavirus	0.49	0.10	27.05	1	0.000	1.64	1.36	1.97
C3. Fear about family member's health due to coronavirus	0.30	0.08	15.22	1	0.000	1.36	1.16	1.58
C4. Fear about stigmatization after illness (coronavirus)	0.16	0.07	5.79	1	0.016	1.18	1.03	1,35
P1. Religious / spiritual inquiries	−0.08	0.12	0.49	1	0.485	0.92	0.729	1.16
E3. Conflicts with family members	0.16	0.10	2.73	1	0.099	1.18	0.97	1.42
E4. Changes in the quality of relationships with family members	−0.19	0.14	1.66	1	0.197	0.83	0.63	1.10
E5. Managing to maintain a basic daily routine	−0.30	0.10	9.10	1	0.003	0.74	0.61	0.90
E7. Financial strain	−0.42	0.09	21.06	1	0.000	0.66	0.553	0.79
K1. The information and use of the internet worry me about the issue regarding the COVID-19	0.22	0.07	8.83	1	0.003	1.24	1.08	1.43
K3. Increase in internet usage time	0.05	0.07	0.51	1	0.477	1.05	0.92	1.19
K4. Changes in the use of social media	0.21	0.15	1.98	1	0.160	0.81	0.60	1.09
Loneliness	0.64	0.11	36.59	1	0.000	1.90	1.54	2.34
Emotion Regulation	0.23	0.14	2.87	1	0.090	1.26	0.96	1.64
Psychological Resilience	−0.10	0.02	28.03	1	0.000	0.903	0.87	0.94
Negative Problem Orientation	0.23	0.09	6.52	1	0.011	1.26	1.06	1.51
Positive Problem Orientation	0.14	0.11	1.78	1	0.182	1.15	0.94	1.42
Constant	−2.60	1.12	5.42	1	0.020	0.07		

*Gender is for, females compared to males; B2. Chronic medical somatic condition is for “yes” response compared to “no”; B4. Caretaker of person from vulnerable group is for “yes” response compared to “no”; B5. Mental health problems in the past are for “yes” response compared to “no”. Odds Ratio = Exp(B)*.

Protective factors found to be improvements in general health status (OR = 0.68, 95 % CI 0.58–0.81, *p* < 0.001), maintaining one's daily routine (OR = 0.74, 95 % CI 0.61–0.90, *p* = 0.003), having a stable economic situation (OR = 0.66, 95 % CI 0.55–0.79, *p* < 0.001), and having good psychological resilience (OR = 0.90, 95 % CI 0.87–0.94, *p* < 0.001).

## Discussion

This study investigates the association between anxiety and sociodemographic and health-related characteristics (e.g., such as suicidality, fear of COVID-19, relationship quality, daily routine, and Internet use) as well as psychological determinants to predict the factors for anxiety, using a representative sample of general Latvian population during the COVID-19 state of emergency. The study sample included 2,608 participants. It is noteworthy, that the prevalence of anxiety in the general Latvian population has not yet been estimated, although there is currently an ongoing population study on the prevalence of mental disorders and suicidality in Latvia (29). The current study found that the prevalence of anxiety was estimated at 15.2%, which is in line with the average prevalence of anxiety disorders in Europe (48). Many studies have suggested that COVID-19 has triggered higher levels of anxiety and distress (8, 9, 18) than the estimated anxiety prevalence rate found in our study. However, it is important to mention that different methodologies and tools have been used across these studies, and high level of anxiety might also depend on the temporal situation and specific events (49, 50). Another important aspect is that individuals who have been isolated and quarantined due to COVID-19 have experienced significant levels of anxiety, anger, confusion, and fear (51). Moreover, at the time of our study, restrictions related to the pandemic in Latvia were much milder than in other Baltic and European countries.

The data analysis revealed that anxiety was 2.44 times more prevalent in females than males. This finding is in accordance with most of the data received from different countries, and indicates that females are at a higher risk of anxiety disorders (7, 52). Although anxiety was more prevalent in the youngest age group, age was not significant in the logistic regression model.

Unsurprisingly, the data indicate that people with pre-existing mental health disorders show higher levels of COVID-19-related anxiety than those with no history of mental health disorders (17, 18). Our study confirmed this finding, as those who had mental health issues in the past were 1.45 times more likely to have anxiety. While some studies have also indicated that individuals with chronic medical conditions are more likely to have anxiety (53), our study found that the presence of a chronic somatic disorder was not a significant predictor. It is noteworthy that we found that the presence of chronic somatic disorders in the general Latvian population was not a risk factor for depression during the state of emergency from March to June 2020 (29). Moreover, in the study on the 12-month prevalence of major depression in Latvia was found that presence of three or more self-reported somatic conditions is related to increased odds of major depression, while presence of one or two somatic disorders is not (54). Moreover, in our study, self-rated better general health was related to decreased odds of having anxiety and served as a protective factor.

The previous literature has addressed that before the pandemic, acute stress was related to suicide ideation in older adults who had severe medical conditions. Moreover, the high risk of suicide during the pandemic has been associated with high levels of perceived stress, depression, and insomnia (24). Our study found that during the state of emergency, the fear of dying, thoughts of harming one's self, and suicide ideation were more prevalent in those who had anxiety. The logistic regression analysis revealed that fear of dying during the state of emergency and suicidality in the past increased the odds of having anxiety and, therefore, were significant predictors of anxiety, but self-harm behavior in the past was not a significant predictor. Fountoulakis et al. (17) developed a model to explain the effect of the pandemic on mental health that is based on the assumption that anxiety develops first and then progresses into depression and then suicidality.

Fears about the COVID-19 pandemic, one's health status, family members, and stigmatization were significantly more prevalent in those who had anxiety and served as predictors to anxiety. The data from previous studies have suggested that the COVID-19 pandemic has contributed to existential fears of infection and death (18). Moreover, the existing research has highlighted the important role of the complex relationship between fear, stress, and anxiety in the development of depression (55).

In our study, a decline in the overall quality of family relationships and increased family conflicts were more prevalent in the participants who had higher anxiety scores. Anxiety was also more prevalent among those who had difficulty in maintaining a basic daily routine. Maintaining a healthy lifestyle to help foster self-efficacy can, therefore, be presented as a protective factor for anxiety (56). The logistic regression model revealed two important factors that played a protective role against anxiety: maintaining a daily routine and having financial stability. These findings are in line with the existing research (57).

The previous studies have indicated a rise in problematic Internet use and overuse by the general population during the pandemic (58). Disordered Internet use generates marked distress, worry, and significant impairment in personal, family, social, educational, and occupational functioning (59). Moreover, Internet browsing about COVID-19, distress related to this information, excessive time spent on the Internet, and increased use of social media have been associated with increased anxiety in the general population during the pandemic (60). Although we found that excessive time spent online and more frequent social media use during the state of emergency was more prevalent among those who had anxiety, the logistic regression analysis revealed that these factors were not significant predictors of anxiety. In our study, excessive worrying about COVID-19 was a significant risk factor for having anxiety (OR = 1.24), yet a change in social media use was not a risk factor, which is in line with a study on interactions between anxiety levels and life habits changes in the general population of Russia (3). We also found that an increase in Internet usage was not a significant predictor.

Loneliness has been identified as a major adverse consequence of the COVID-19 pandemic. The previous studies have reported that when people are isolated or lonely, they become significantly more vulnerable to anxiety (61, 62). In our study, those who had experienced loneliness were 1.90 times more likely to risk having anxiety. This result indicates that anxiety can be predicted when people have low psychological resilience. Our results support the recent studies during the COVID-19 pandemic that show that having a lower psychological resilience score indicates a higher level of anxiety (13). Our data also show that anxiety can be predicted by having a negative orientation in problem-solving during the pandemic.

A major strength of our study is that it includes a large representative sample of the general Latvian population, which allows for both estimations and determinants of anxiety at the national level. Our results also highlight the importance of supporting those who are at risk to alleviate suffering in the instance of future possible lockdowns, and emphasize that groups that already had poor mental health before the pandemic are at risk both during and after the pandemic. These findings show the importance of providing the community with the necessary psychological support to reduce anxiety. In addition to focusing on the negative effects, it is very important to develop prevention and intervention measures that aim at thriving, so as to reduce harm and achieve positive results (18).

This study has several practical implications. Our findings can help develop future strategies for managing psychological support for segments of the population who are at risk. Our results indicate that the following measures could be implemented: (1) improve the recognition of anxiety and other mental disorders at the primary-care level and provide general practitioners with advice and consultations from mental health specialists; (2) use a variety of communication channels (e.g., infographics, social media, school websites, etc.) to inform the target group about simple, realistic, effective, and evidence-based self-help strategies for mental health prevention, and promote and strengthen psychological resilience techniques; and (3) enable collaboration between psychiatrists, psychologists, and policymakers to develop effective interventions and implementation strategies to strengthen the psychological resilience of the Latvian population.

## Conclusion

This study examines the association between various factors and anxiety, and identifies the predicting factors for anxiety using a representative sample of the general Latvian population during the COVID-19 pandemic. We identified the following predictors for anxiety: being female, having mental health problems in the past, suicidality, having fears about one's health status due to COVID-19, fear of stigmatization if infected with COVID-19, worrying about information on the Internet, loneliness, and having negative problem orientation. Protective factors were also identified (improvements in general health status, maintaining one's daily routine, having a stable economic situation, and having good psychological resilience). These findings confirm previous recommendations by other authors on the need for proactive intervention to protect the mental health of the population, but especially of vulnerable groups (17).

### Limitations

The results of current paper must be considered in the context of some limitations. Our cross-sectional study did not allow us to make any causal interferences. Therefore, further longitudinal studies could provide more information on causal relationships. An important limitation is that invitations were sent to potential respondents *via* e-mail. For that reason certain groups of the Latvian populations probably were less likely to fill in the questionnaire. Another important limitation that may have influenced the results is the use of self-report measures and scales. For example, anxiety symptoms were measured using a self-reported questionnaire which may have brought bias to an overestimation or underestimation of the prevalence of observed pathology. Moreover, there is no clinical verification of anxiety disorders. Finally, recall bias may have influenced some measures, such as report of existing chronic somatic disorders. It should be noted that Latvian population speak Latvian or Russian and the preparation phase of the study was limited in time, therefore it was not possible to validate the measures used in the COMET-G study. Voluntary recruitment can also lead to so-called non-response bias, where non-respondents may have different characteristics than survey respondents. In the present study it was impossible to identify whether the non-participants were significantly different from the sample of the survey respondents, and this is one major limitation of our study. It is noteworthy to mention that as a part of the study was international, the use of a single protocol was critical. It is also important to state that the data were collected in July 2020, in the period, when number of COVID-19 cases in Latvia was low. Moreover, during the state of emergency from March to June 2020, the COVID-19 restrictions were noticeably milder than in other Baltic and European countries. Finally, use of highly related variables in logistic models may affect significance. It would be worthwhile to conduct a similar study in the future to investigate the long-term outcome and the long-term impact of the pandemics on mental-health of the Latvian population because of more strict COVID-19 restrictions, and significantly increased rates of the cases of infected people and the death rate. Finally, the lack of baseline data concerning anxiety and related factors before the pandemic did not allow us to make any comparisons.

## Data Availability Statement

The raw data supporting the conclusions of this article will be made available by the authors, without undue reservation.

## Ethics Statement

The studies involving human participants were reviewed and approved by Ethics Committee of Research in Riga Stradins University. The first page of the online questionnaire included the declaration of voluntarily consent for participation. The patients/participants provided their written informed consent to participate in this study.

## Author Contributions

JV contributed with development of the conception and design of the study and he is a national coordinator of COMET-G project, participated in the translation of the protocol to Latvian and Russian languages, and wrote the first draft of the manuscript. VP contributed with the development of the conception and design of the study, development of the questions for the questionnaire, was responsible for the translation of the developed questionnaire into Russian, was responsible for the statistical data analysis and the result part of the manuscript, interpreted the data, and participated in writing the manuscript. KM contributed with development of the conception and design of the study, development of the questions for the questionnaire, and participated in writing the manuscript. JK contributed with development of the conception and design of the study, was responsible for the translation of the developed questionnaire into Russian, and participated in writing the manuscript. IK participated in writing the manuscript. DS coordinator of the COMET-G project. KF principal investigator of the COMET-G project and development of the study protocol. ER contributed with development of the conception and design of the study, is a national coordinator of the COMET-G project, and participated in writing the manuscript. All authors participated in interpreting the data and developing further stages and the final version of the paper.

## Funding

The study is a part of a National Research Programme to Mitigate Consequences of COVID-19 approved by the Latvian Council of Science (VPP COVID_2020/1-0011).

## Conflict of Interest

The authors declare that the research was conducted in the absence of any commercial or financial relationships that could be construed as a potential conflict of interest.

## Publisher's Note

All claims expressed in this article are solely those of the authors and do not necessarily represent those of their affiliated organizations, or those of the publisher, the editors and the reviewers. Any product that may be evaluated in this article, or claim that may be made by its manufacturer, is not guaranteed or endorsed by the publisher.
